# [μ-Bis(diphenyl­phosphan­yl)methane]­tricarbon­yl(μ-*p*-toluene­sulfonyl­meth­yl isocyanato)(triphenyl­phosphane)ironplatinum(*Fe*—*Pt*)

**DOI:** 10.1107/S1600536812004023

**Published:** 2012-02-24

**Authors:** Isabelle Jourdain, Michael Knorr, Stephan G. Koller, Carsten Strohmann

**Affiliations:** a Institut UTINAM UMR 6213 CNRS, Université de Franche-Comté, 16 Route de Gray, 25030 Besançon Cedex, France; b Anorganische Chemie, Technische Universität Dortmund, Otto-Hahn-Strasse 6, D-44227 Dortmund, Germany

## Abstract

The title compound, [FePt(C_9_H_9_NO_2_S)(C_18_H_15_P)(C_25_H_22_P_2_)(CO)_3_], represents a rare example of an isonitrile-bridged heterobimetallic complex (here Pt and Fe) and is an inter­esting precursor for the preparation of heterodinuclear μ-amino­carbyne complexes, since the basic imine-type N atom of the μ_2_-C=N–*R* ligand readily undergoes addition with various electrophiles to afford iminium-like salts. In the crystal, the almost symmetrically bridging μ_2_-C=N-*R* ligand (neglecting the different atomic radii of Fe and Pt) is strongly bent towards the Fe(CO)_3_ fragment, with a C=N-*R* angle of only 121.1 (4)°.

## Related literature

The title compound was first prepared in 2002; see: Knorr *et al.* (2002 ▶). Concerning the *N*-protonation and *N*-alkyl­ation of related [(OC)_3_Fe(μ-C=N-*R*)(μ-dppm)Pt(PPh_3_)] complexes, see: Knorr *et al.* (1993 ▶). For a structural comparison with [(OC)_3_Fe(μ-C=N-*o*-anis­yl)(μ-dppm)Pt(PPh_3_)], see: Knorr & Strohmann (2000 ▶). For a structural comparison with [(OC)_4_W(μ-C=N-CH_2_SO_2_-*p*-tol­yl)(μ-dppm)Pt(PPh_3_)], see: Knorr *et al.* (2003 ▶). For a structural comparison with [(OC)_3_Fe(μ-C=O)(μ-dppm)Pt(PPh_3_)], see: Fontaine *et al.* (1988 ▶). For a structural comparision with [ClPt(μ-dppm)_2_(μ-C=NMe)Ni(CNMe)]Cl, see Ratliff *et al.* (1990 ▶). For a structural comparision with [Cp*FeCNPh(μ-C=NPh)(μ-SEt)PdCl(PPh_3_)]PF_6_, see Chen *et al.* (2010 ▶). For a structural comparision with [(EtNC)_3_Fe(μ-C=NEt)_3_Fe(CNEt)_3_], see Basset *et al.* (1981 ▶).
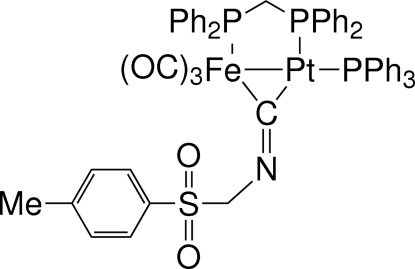


## Experimental

### 

#### Crystal data

[FePt(C_9_H_9_NO_2_S)(C_18_H_15_P)(C_25_H_22_P_2_)(CO)_3_]
*M* *_r_* = 1176.84
Monoclinic, 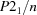

*a* = 14.360 (4) Å
*b* = 18.855 (6) Å
*c* = 17.919 (5) Å
β = 96.50 (3)°
*V* = 4820 (2) Å^3^
*Z* = 4
Mo *K*α radiation
μ = 3.39 mm^−1^
*T* = 173 K
0.40 × 0.20 × 0.20 mm

#### Data collection

Stoe IPDS diffractometer
Absorption correction: numerical (*FACEIT* in *IPDS*; Stoe & Cie, 1999 ▶) *T*_min_ = 0.344, *T*_max_ = 0.550
38854 measured reflections
9346 independent reflections
7344 reflections with *I* > 2σ(*I*)
*R*_int_ = 0.052

#### Refinement

*R*[*F* ^2^ > 2σ(*F* ^2^)] = 0.039
*wR*(*F* ^2^) = 0.090
*S* = 1.01
9346 reflections
605 parameters
H-atom parameters constrained
Δρ_max_ = 1.26 e Å^−3^
Δρ_min_ = −1.37 e Å^−3^

### 

Data collection: *EXPOSE* in *IPDS* (Stoe & Cie, 1999 ▶); cell refinement: *CELL* in *IPDS*; data reduction: *INTEGRATE* in *IPDS*; program(s) used to solve structure: *SHELXS97* (Sheldrick, 2008 ▶); program(s) used to refine structure: *SHELXL97* (Sheldrick, 2008 ▶); molecular graphics: *ORTEP-3* (Farrugia, 1997 ▶); software used to prepare material for publication: *WinGX* (Farrugia, 1999 ▶).

## Supplementary Material

Crystal structure: contains datablock(s) I, global. DOI: 10.1107/S1600536812004023/fi2121sup1.cif

Structure factors: contains datablock(s) I. DOI: 10.1107/S1600536812004023/fi2121Isup2.hkl

Additional supplementary materials:  crystallographic information; 3D view; checkCIF report

## Figures and Tables

**Table:** 

C1—Fe	1.778 (5)
C2—Fe	1.753 (6)
C3—Fe	1.792 (5)
C4—Pt	1.987 (6)
C4—Fe	2.003 (5)
Fe—P1	2.2389 (14)
Fe—Pt	2.5555 (9)
P2—Pt	2.3278 (13)
P3—Pt	2.2680 (13)

**Table:** 

N1—C4—Pt	139.4 (4)
N1—C4—Fe	140.7 (4)
Pt—C4—Fe	79.6 (2)

**Fig. 1.**
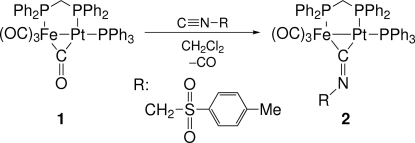
Synthesis of the heterobimetallic µ2-C═NR isocyanide complex 2.

**Fig. 2.**
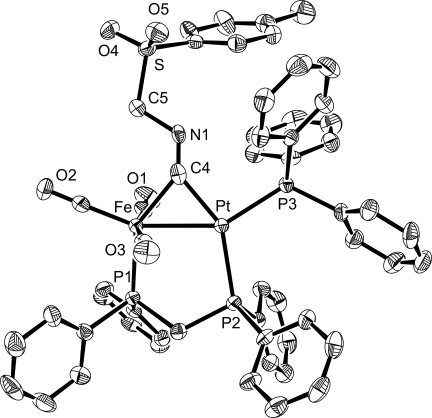
Thermal ellipsoid plot of 2 at the 50% probability level (H atoms were omitted for clarity).

## supplementary crystallographic information

### Comment

Isonitriles, also called isocyanides (RNC) are often employed as ligands in coordination chemistry, since steric and electronic variation of the group *R* permits fine-tuning of the properties of a metal complex. In addition to the innumerable examples of mononuclear isonitrile complexes, many heterobimetallic complexes and higher nuclearity clusters bearing *terminal* isonitrile ligands have now been documented. However, polymetallic systems containing an isonitrile bridge between *two different* metal centers are still extremely scarce. Crystallograpically characterized examples include [ClPt(µ-dppm)_2_(µ-C═NMe)Ni(CNMe)]Cl and [Cp*FeCNPh(µ-C═NPh)(µ-SEt)PdCl(PPh_3_)][PF_6_] (Ratliff *et al.*, 1990; Chen *et al.*, 2010). This paucity is quite surprising, since metal-bound CO, which acts like CNR as a σ-donor and π-acceptor ligand, readily adopts a µ_2_-C═O bonding mode in heterometallic systems, where the metal centres are connected through a metal–metal bond. In the context of our interest on the activation of small unsaturated organic molecules such as olefins, alkynes and isocyanides, we have recently reported the synthesis of [(OC)_3_Fe(µ-C═N-CH_2_SO_2_*p*-tolyl)(µ-dppm)Pt(PPh_3_)] by reduction of [(OC)_4_Fe(µ-dppm)PtCl_2_(C≡N-CH_2_SO_2_*p*-tolyl)] in the presence of PPh_3_. Alternatively, the title compound is readily prepared by substitution of the bridging carbonyl group of [(OC)_3_Fe(µ-C═O)(µ-dppm)Pt(PPh_3_)] (1) by addition of an equimolar amount of the isocyanide according Fig. 1.

The title compound 2 crystallizes from CH_2_Cl_2_/hexane in the monoclinic crystal system, space group *P*2_1_/n. As shown in Fig. 2, the iron and platinum centres are linked by a dppm-bridge and a metal–metal bond, whose Fe–Pt separation of 2.5555 (9) Å is quite similar with that of the precursor 1 [Fe–Pt 2.579 (4) Å]. Also the other structural features are very reminiscent to the molecular structure of the latter compound. The bridging isonitrile ligand is symmetrically situated between the metals, with Fe—C(4) and Pt—C(4) distances of 2.003 (5) and 1.988 (6) Å, respectively. The length of the C(4)—N double bond of 1.180 (7) Å is somewhat shorter than that of the W—Pt complex [(OC)_4_W(µ-C═N-CH_2_SO_2_*p*-tolyl)(µ-dppm)Pt(PPh_3_)] [1.229 (12) Å] (Knorr *et al.*, 2003) and matches with that of [(OC)_3_Fe(µ-C═N-*o*-anisyl)(µ-dppm)Pt(PPh_3_)] [1.191 (12) Å] (Knorr & Strohmann, 2000). The C(4)—N(1)—C(5) angle of 121.1 (4)° is extremely bent, the tosylmethyl group being oriented toward the Fe(CO)_3_ moiety. A comparable inclination with an average C—N—C angle of 123.1° was found for the µ-C═N-Et ligands of [(EtNC)_3_Fe(µ-C═N-Et)_3_Fe(CNEt)_3_], (Basset *et al.*, 1981) whereas for the most part of structurally characterized bent µ-CNR systems C—N—C angles in the range between 130–133° have been documented.

### Experimental

Solid *p*-tosylmethylisocyanide (98 mg, 0.5 mmol) was added in three portions to a solution of [(OC)_3_Fe(µ-CO)(µ-dppm)Pt(PPh_3_)] (1) (505 mg, 0.5 mmol) in 10 ml of CH_2_Cl_2_. The orange reaction mixture was stirred at room temperature for 30 min and then concentrated to 5 ml under reduced pressure. Addition of a hexane layer gave after storage for 2 d at 5°C in a refrigerator air-stable yellow–orange crystals (459 mg, 78% yield). Characterization data have been previously described in the literature (Knorr *et al.*, 2002).

### Refinement

All H atoms were refined in geometrical idealized position using the riding model [aromatic C: C—H = 0.95 Å, *U*_iso_(H) = 1.2*U*_eq_(C); CH_2_: C—H = 0.99 Å, *U*_iso_(H) = 1.2*U*_eq_(C); CH_3_: C—H = 0.98 Å, *U*_iso_(H) = 1.5*U*_eq_(C)].

### Crystal data

**Table:**

[FePt(C_9_H_9_NO_2_S)(C_18_H_15_P)(C_25_H_22_P_2_)(CO)_3_]	*F*(000) = 2352
*M**_r_* = 1176.84	*D*_x_ = 1.622 Mg m^−^^3^
Monoclinic, *P*2_1_/*n*	Mo *K*α radiation, λ = 0.71073 Å
Hall symbol: -P 2yn	Cell parameters from 47431 reflections
*a* = 14.360 (4) Å	θ = 2.2–28.3°
*b* = 18.855 (6) Å	µ = 3.39 mm^−^^1^
*c* = 17.919 (5) Å	*T* = 173 K
β = 96.50 (3)°	Needle, yellow-orange
*V* = 4820 (2) Å^3^	0.40 × 0.20 × 0.20 mm
*Z* = 4	

### Data collection

**Table:**

Stoe IPDS diffractometer	9346 independent reflections
Radiation source: fine-focus sealed tube	7344 reflections with *I* > 2σ(*I*)
Graphite monochromator	*R*_int_ = 0.052
φ scans	θ_max_ = 26.0°, θ_min_ = 2.2°
Absorption correction: numerical (FACEIT in *IPDS*; Stoe & Cie, 1999)	*h* = −17→17
*T*_min_ = 0.344, *T*_max_ = 0.550	*k* = −23→23
38854 measured reflections	*l* = −21→21

### Refinement

**Table:**

Refinement on *F*^2^	Primary atom site location: structure-invariant direct methods
Least-squares matrix: full	Secondary atom site location: difference Fourier map
*R*[*F*^2^ > 2σ(*F*^2^)] = 0.039	Hydrogen site location: inferred from neighbouring sites
*wR*(*F*^2^) = 0.090	H-atom parameters constrained
*S* = 1.01	*w* = 1/[σ^2^(*F*_o_^2^) + (0.0547*P*)^2^] where *P* = (*F*_o_^2^ + 2*F*_c_^2^)/3
9346 reflections	(Δ/σ)_max_ = 0.002
605 parameters	Δρ_max_ = 1.26 e Å^−^^3^
0 restraints	Δρ_min_ = −1.37 e Å^−^^3^

### Special details

**Table:**

Geometry. All e.s.d.'s (except the e.s.d. in the dihedral angle between two l.s. planes) are estimated using the full covariance matrix. The cell e.s.d.'s are taken into account individually in the estimation of e.s.d.'s in distances, angles and torsion angles; correlations between e.s.d.'s in cell parameters are only used when they are defined by crystal symmetry. An approximate (isotropic) treatment of cell e.s.d.'s is used for estimating e.s.d.'s involving l.s. planes.
Refinement. Refinement of *F*^2^ against ALL reflections. The weighted *R*-factor *wR* and goodness of fit *S* are based on *F*^2^, conventional *R*-factors *R* are based on *F*, with *F* set to zero for negative *F*^2^. The threshold expression of *F*^2^ > σ(*F*^2^) is used only for calculating *R*-factors(gt) *etc*. and is not relevant to the choice of reflections for refinement. *R*-factors based on *F*^2^ are statistically about twice as large as those based on *F*, and *R*-factors based on ALL data will be even larger.

### Fractional atomic coordinates and isotropic or equivalent isotropic displacement parameters (Å^2^)

**Table:**

	*x*	*y*	*z*	*U*_iso_*/*U*_eq_	
C1	0.5805 (4)	0.3172 (3)	0.6668 (3)	0.0320 (12)	
C2	0.5076 (4)	0.2169 (3)	0.5794 (3)	0.0326 (12)	
C3	0.4134 (3)	0.1848 (2)	0.6959 (3)	0.0274 (11)	
C4	0.4066 (3)	0.3216 (2)	0.6688 (3)	0.0286 (11)	
C5	0.3800 (4)	0.3612 (3)	0.5460 (3)	0.0322 (12)	
H5A	0.3650	0.3136	0.5244	0.039*	
H5B	0.4468	0.3710	0.5415	0.039*	
C6	0.3237 (3)	0.5044 (2)	0.5453 (3)	0.0278 (11)	
C7	0.2672 (4)	0.5181 (3)	0.6014 (3)	0.0335 (12)	
H7	0.2242	0.4833	0.6148	0.040*	
C8	0.2741 (4)	0.5826 (3)	0.6376 (3)	0.0384 (13)	
H8	0.2362	0.5919	0.6766	0.046*	
C9	0.3357 (4)	0.6341 (3)	0.6176 (4)	0.0417 (14)	
C10	0.3926 (4)	0.6188 (3)	0.5623 (4)	0.0478 (16)	
H10	0.4360	0.6535	0.5492	0.057*	
C11	0.3874 (4)	0.5545 (3)	0.5262 (4)	0.0371 (13)	
H11	0.4271	0.5444	0.4885	0.045*	
C12	0.3380 (5)	0.7052 (3)	0.6543 (4)	0.061 (2)	
H12A	0.2748	0.7179	0.6653	0.092*	
H12B	0.3603	0.7407	0.6205	0.092*	
H12C	0.3803	0.7038	0.7012	0.092*	
C13	0.7264 (3)	0.2207 (2)	0.7703 (3)	0.0258 (10)	
C14	0.7691 (3)	0.2306 (3)	0.8422 (3)	0.0289 (11)	
H14	0.7373	0.2183	0.8841	0.035*	
C15	0.8591 (4)	0.2588 (3)	0.8534 (3)	0.0356 (12)	
H15	0.8894	0.2649	0.9030	0.043*	
C16	0.9045 (3)	0.2779 (3)	0.7926 (3)	0.0351 (13)	
H16	0.9654	0.2982	0.8004	0.042*	
C17	0.8622 (3)	0.2678 (3)	0.7209 (3)	0.0353 (12)	
H17	0.8940	0.2801	0.6790	0.042*	
C18	0.7727 (3)	0.2395 (3)	0.7099 (3)	0.0321 (12)	
H18	0.7429	0.2330	0.6603	0.038*	
C19	0.6353 (3)	0.0928 (2)	0.7231 (3)	0.0274 (11)	
C20	0.5854 (4)	0.0593 (3)	0.6636 (3)	0.0348 (12)	
H20	0.5368	0.0838	0.6339	0.042*	
C21	0.6056 (5)	−0.0103 (3)	0.6465 (4)	0.0500 (16)	
H21	0.5712	−0.0334	0.6050	0.060*	
C22	0.6751 (5)	−0.0455 (3)	0.6898 (5)	0.0545 (19)	
H22	0.6884	−0.0935	0.6785	0.065*	
C23	0.7252 (5)	−0.0133 (3)	0.7485 (4)	0.0550 (18)	
H23	0.7741	−0.0382	0.7775	0.066*	
C24	0.7055 (4)	0.0556 (3)	0.7661 (4)	0.0416 (14)	
H24	0.7401	0.0780	0.8080	0.050*	
C25	0.5661 (3)	0.1660 (3)	0.8403 (3)	0.0243 (10)	
H25A	0.6207	0.1538	0.8768	0.029*	
H25B	0.5245	0.1240	0.8352	0.029*	
C26	0.4384 (3)	0.1922 (3)	0.9461 (3)	0.0258 (10)	
C27	0.4593 (4)	0.1232 (3)	0.9700 (3)	0.0360 (12)	
H27	0.5104	0.0988	0.9524	0.043*	
C28	0.4060 (4)	0.0900 (3)	1.0193 (3)	0.0408 (13)	
H28	0.4195	0.0424	1.0342	0.049*	
C29	0.3342 (4)	0.1251 (3)	1.0467 (3)	0.0405 (14)	
H29	0.2989	0.1024	1.0815	0.049*	
C30	0.3131 (4)	0.1930 (3)	1.0241 (4)	0.0445 (14)	
H30	0.2624	0.2171	1.0427	0.053*	
C31	0.3651 (4)	0.2269 (3)	0.9740 (3)	0.0353 (12)	
H31	0.3502	0.2742	0.9588	0.042*	
C32	0.5933 (3)	0.2874 (2)	0.9386 (3)	0.0250 (10)	
C33	0.6381 (4)	0.2575 (3)	1.0035 (3)	0.0308 (11)	
H33	0.6184	0.2126	1.0197	0.037*	
C34	0.7103 (4)	0.2922 (3)	1.0446 (3)	0.0391 (13)	
H34	0.7413	0.2708	1.0886	0.047*	
C35	0.7381 (4)	0.3582 (3)	1.0219 (4)	0.0435 (14)	
H35	0.7881	0.3824	1.0506	0.052*	
C36	0.6935 (4)	0.3892 (3)	0.9578 (4)	0.0417 (14)	
H36	0.7121	0.4348	0.9426	0.050*	
C37	0.6217 (4)	0.3534 (3)	0.9161 (3)	0.0325 (12)	
H37	0.5914	0.3743	0.8715	0.039*	
C38	0.3223 (3)	0.4062 (2)	0.9132 (3)	0.0265 (10)	
C39	0.3962 (4)	0.4227 (3)	0.9664 (3)	0.0350 (12)	
H39	0.4563	0.4320	0.9513	0.042*	
C40	0.3833 (4)	0.4259 (3)	1.0420 (3)	0.0448 (14)	
H40	0.4340	0.4392	1.0780	0.054*	
C41	0.2973 (4)	0.4098 (3)	1.0652 (3)	0.0439 (14)	
H41	0.2893	0.4101	1.1171	0.053*	
C42	0.2239 (4)	0.3935 (3)	1.0130 (4)	0.0422 (14)	
H42	0.1642	0.3837	1.0287	0.051*	
C43	0.2354 (4)	0.3912 (3)	0.9368 (3)	0.0336 (12)	
H43	0.1839	0.3793	0.9010	0.040*	
C44	0.4038 (3)	0.4811 (3)	0.7998 (3)	0.0276 (11)	
C45	0.3826 (4)	0.5437 (3)	0.8353 (3)	0.0345 (12)	
H45	0.3366	0.5439	0.8695	0.041*	
C46	0.4280 (4)	0.6050 (3)	0.8210 (4)	0.0449 (15)	
H46	0.4134	0.6476	0.8453	0.054*	
C47	0.4946 (4)	0.6057 (3)	0.7718 (4)	0.0496 (17)	
H47	0.5259	0.6487	0.7625	0.060*	
C48	0.5162 (4)	0.5447 (3)	0.7362 (3)	0.0402 (14)	
H48	0.5620	0.5450	0.7019	0.048*	
C49	0.4711 (3)	0.4836 (3)	0.7506 (3)	0.0345 (12)	
H49	0.4864	0.4413	0.7260	0.041*	
C50	0.2270 (3)	0.4062 (3)	0.7653 (3)	0.0260 (10)	
C51	0.1876 (4)	0.3497 (3)	0.7237 (3)	0.0333 (12)	
H51	0.2232	0.3077	0.7195	0.040*	
C52	0.0988 (4)	0.3536 (3)	0.6889 (3)	0.0383 (13)	
H52	0.0728	0.3143	0.6606	0.046*	
C53	0.0459 (4)	0.4139 (4)	0.6941 (4)	0.0458 (15)	
H53	−0.0163	0.4163	0.6698	0.055*	
C54	0.0837 (4)	0.4700 (3)	0.7342 (4)	0.0439 (15)	
H54	0.0476	0.5118	0.7376	0.053*	
C55	0.1739 (3)	0.4670 (3)	0.7702 (3)	0.0342 (12)	
H55	0.1995	0.5066	0.7981	0.041*	
Fe	0.50291 (4)	0.24438 (3)	0.67242 (4)	0.02043 (14)	
N1	0.3652 (3)	0.3610 (2)	0.6259 (2)	0.0242 (9)	
O1	0.6270 (3)	0.3649 (2)	0.6603 (3)	0.0487 (11)	
O2	0.5088 (3)	0.1990 (2)	0.5180 (2)	0.0533 (12)	
O3	0.3565 (3)	0.1450 (2)	0.7070 (2)	0.0432 (10)	
O4	0.3496 (3)	0.4347 (2)	0.4249 (2)	0.0422 (9)	
O5	0.2135 (3)	0.40500 (19)	0.4911 (2)	0.0398 (9)	
P1	0.60807 (8)	0.18336 (6)	0.74887 (7)	0.0215 (2)	
P2	0.50237 (8)	0.23947 (6)	0.87895 (7)	0.0232 (3)	
P3	0.34487 (8)	0.39739 (6)	0.81580 (7)	0.0232 (3)	
Pt	0.426732 (12)	0.303384 (9)	0.778673 (10)	0.02230 (6)	
S	0.30918 (9)	0.42593 (6)	0.49380 (7)	0.0290 (3)	

### Atomic displacement parameters (Å^2^)

**Table:**

	*U*^11^	*U*^22^	*U*^33^	*U*^12^	*U*^13^	*U*^23^
C1	0.028 (3)	0.031 (3)	0.036 (3)	0.010 (2)	0.002 (2)	0.000 (2)
C2	0.033 (3)	0.032 (3)	0.033 (3)	0.012 (2)	0.005 (2)	0.003 (2)
C3	0.029 (2)	0.022 (2)	0.030 (3)	0.003 (2)	−0.002 (2)	0.000 (2)
C4	0.026 (2)	0.025 (2)	0.036 (3)	−0.0054 (19)	0.012 (2)	−0.007 (2)
C5	0.034 (3)	0.033 (3)	0.030 (3)	0.010 (2)	0.005 (2)	−0.001 (2)
C6	0.029 (2)	0.024 (2)	0.029 (3)	0.0041 (19)	−0.003 (2)	0.005 (2)
C7	0.040 (3)	0.027 (3)	0.035 (3)	0.003 (2)	0.007 (2)	0.004 (2)
C8	0.048 (3)	0.035 (3)	0.031 (3)	0.010 (2)	0.004 (3)	−0.004 (2)
C9	0.045 (3)	0.032 (3)	0.045 (4)	0.002 (2)	−0.008 (3)	−0.006 (3)
C10	0.040 (3)	0.033 (3)	0.071 (5)	−0.006 (2)	0.011 (3)	−0.011 (3)
C11	0.033 (3)	0.031 (3)	0.049 (4)	0.002 (2)	0.014 (3)	0.000 (2)
C12	0.071 (5)	0.039 (4)	0.069 (5)	−0.002 (3)	−0.015 (4)	−0.018 (3)
C13	0.025 (2)	0.025 (2)	0.028 (3)	0.0085 (19)	0.004 (2)	0.001 (2)
C14	0.028 (3)	0.033 (3)	0.026 (3)	0.004 (2)	0.004 (2)	0.002 (2)
C15	0.028 (3)	0.043 (3)	0.034 (3)	0.005 (2)	−0.004 (2)	0.000 (2)
C16	0.020 (2)	0.032 (3)	0.053 (4)	0.003 (2)	0.003 (2)	0.006 (2)
C17	0.024 (2)	0.040 (3)	0.043 (4)	0.002 (2)	0.007 (2)	0.011 (3)
C18	0.032 (3)	0.035 (3)	0.030 (3)	0.003 (2)	0.005 (2)	0.007 (2)
C19	0.032 (3)	0.021 (2)	0.030 (3)	0.0021 (19)	0.010 (2)	0.003 (2)
C20	0.041 (3)	0.028 (3)	0.039 (3)	0.001 (2)	0.015 (2)	−0.002 (2)
C21	0.068 (4)	0.031 (3)	0.054 (4)	−0.006 (3)	0.019 (3)	−0.006 (3)
C22	0.069 (4)	0.018 (3)	0.082 (6)	0.005 (3)	0.037 (4)	0.003 (3)
C23	0.066 (4)	0.037 (3)	0.064 (5)	0.024 (3)	0.017 (4)	0.013 (3)
C24	0.052 (3)	0.032 (3)	0.041 (4)	0.007 (2)	0.008 (3)	0.005 (2)
C25	0.021 (2)	0.029 (2)	0.024 (3)	−0.0006 (18)	0.0067 (19)	0.004 (2)
C26	0.028 (2)	0.032 (2)	0.018 (2)	−0.004 (2)	0.0025 (19)	0.004 (2)
C27	0.041 (3)	0.039 (3)	0.028 (3)	−0.001 (2)	0.006 (2)	0.011 (2)
C28	0.052 (3)	0.040 (3)	0.031 (3)	−0.010 (3)	0.004 (3)	0.007 (2)
C29	0.044 (3)	0.052 (4)	0.028 (3)	−0.020 (3)	0.012 (2)	0.000 (3)
C30	0.039 (3)	0.058 (4)	0.040 (4)	−0.004 (3)	0.020 (3)	−0.007 (3)
C31	0.042 (3)	0.037 (3)	0.028 (3)	0.004 (2)	0.014 (2)	0.003 (2)
C32	0.026 (2)	0.027 (2)	0.022 (3)	0.0050 (18)	0.0029 (19)	−0.0019 (19)
C33	0.034 (3)	0.032 (3)	0.026 (3)	0.001 (2)	0.000 (2)	−0.003 (2)
C34	0.041 (3)	0.041 (3)	0.033 (3)	0.005 (2)	−0.003 (2)	−0.005 (2)
C35	0.039 (3)	0.045 (3)	0.046 (4)	−0.007 (3)	0.001 (3)	−0.014 (3)
C36	0.043 (3)	0.029 (3)	0.055 (4)	−0.009 (2)	0.013 (3)	−0.001 (3)
C37	0.035 (3)	0.030 (3)	0.033 (3)	0.003 (2)	0.007 (2)	0.004 (2)
C38	0.032 (3)	0.024 (2)	0.024 (3)	0.0026 (19)	0.008 (2)	−0.003 (2)
C39	0.035 (3)	0.040 (3)	0.030 (3)	0.001 (2)	0.007 (2)	0.001 (2)
C40	0.057 (4)	0.046 (3)	0.030 (3)	−0.006 (3)	0.001 (3)	0.000 (3)
C41	0.064 (4)	0.045 (3)	0.025 (3)	0.000 (3)	0.016 (3)	0.000 (3)
C42	0.048 (3)	0.042 (3)	0.042 (4)	−0.006 (3)	0.028 (3)	−0.006 (3)
C43	0.033 (3)	0.037 (3)	0.031 (3)	−0.005 (2)	0.008 (2)	−0.001 (2)
C44	0.026 (2)	0.027 (2)	0.028 (3)	−0.0036 (19)	−0.003 (2)	0.006 (2)
C45	0.035 (3)	0.032 (3)	0.037 (3)	−0.002 (2)	0.004 (2)	−0.005 (2)
C46	0.057 (4)	0.028 (3)	0.048 (4)	−0.008 (3)	0.000 (3)	−0.006 (3)
C47	0.049 (4)	0.043 (3)	0.054 (4)	−0.021 (3)	−0.007 (3)	0.010 (3)
C48	0.035 (3)	0.047 (3)	0.040 (4)	−0.012 (2)	0.007 (2)	0.011 (3)
C49	0.030 (3)	0.036 (3)	0.038 (3)	−0.002 (2)	0.006 (2)	0.003 (2)
C50	0.028 (2)	0.030 (2)	0.022 (3)	0.0033 (19)	0.009 (2)	0.005 (2)
C51	0.033 (3)	0.037 (3)	0.031 (3)	−0.004 (2)	0.009 (2)	−0.005 (2)
C52	0.027 (3)	0.051 (3)	0.037 (3)	−0.006 (2)	0.005 (2)	−0.008 (3)
C53	0.029 (3)	0.067 (4)	0.040 (4)	0.000 (3)	−0.001 (2)	0.008 (3)
C54	0.036 (3)	0.040 (3)	0.057 (4)	0.013 (2)	0.011 (3)	0.008 (3)
C55	0.033 (3)	0.030 (3)	0.041 (3)	0.005 (2)	0.008 (2)	0.000 (2)
Fe	0.0214 (3)	0.0206 (3)	0.0196 (4)	0.0025 (2)	0.0036 (3)	0.0011 (3)
N1	0.0178 (18)	0.029 (2)	0.027 (2)	0.0020 (16)	0.0059 (16)	−0.0072 (18)
O1	0.037 (2)	0.030 (2)	0.080 (3)	−0.0058 (17)	0.014 (2)	0.010 (2)
O2	0.065 (3)	0.068 (3)	0.026 (2)	0.027 (2)	0.004 (2)	−0.010 (2)
O3	0.037 (2)	0.040 (2)	0.054 (3)	−0.0118 (18)	0.0074 (19)	0.0078 (19)
O4	0.063 (3)	0.039 (2)	0.025 (2)	0.0075 (18)	0.0069 (19)	0.0057 (16)
O5	0.035 (2)	0.036 (2)	0.046 (3)	−0.0014 (16)	−0.0060 (17)	−0.0035 (18)
P1	0.0228 (6)	0.0216 (6)	0.0203 (6)	0.0023 (4)	0.0038 (5)	0.0015 (5)
P2	0.0247 (6)	0.0259 (6)	0.0193 (7)	0.0036 (5)	0.0041 (5)	0.0024 (5)
P3	0.0234 (6)	0.0238 (6)	0.0230 (7)	0.0032 (5)	0.0056 (5)	0.0005 (5)
Pt	0.02377 (10)	0.02391 (10)	0.01969 (10)	0.00530 (7)	0.00456 (6)	0.00226 (8)
S	0.0368 (7)	0.0261 (6)	0.0231 (7)	0.0014 (5)	−0.0014 (5)	0.0024 (5)

### Geometric parameters (Å, º)

**Table:**

C1—O1	1.133 (6)	C28—C29	1.362 (9)
C1—Fe	1.778 (5)	C28—H28	0.9500
C2—O2	1.153 (7)	C29—C30	1.366 (9)
C2—Fe	1.753 (6)	C29—H29	0.9500
C3—O3	1.143 (6)	C30—C31	1.387 (8)
C3—Fe	1.792 (5)	C30—H30	0.9500
C4—N1	1.180 (7)	C31—H31	0.9500
C4—Pt	1.987 (6)	C32—C37	1.383 (7)
C4—Fe	2.003 (5)	C32—C33	1.384 (7)
C5—N1	1.471 (7)	C32—P2	1.830 (5)
C5—S	1.785 (5)	C33—C34	1.368 (7)
C5—H5A	0.9900	C33—H33	0.9500
C5—H5B	0.9900	C34—C35	1.383 (8)
C6—C11	1.385 (7)	C34—H34	0.9500
C6—C7	1.386 (8)	C35—C36	1.380 (9)
C6—S	1.744 (5)	C35—H35	0.9500
C7—C8	1.376 (7)	C36—C37	1.380 (8)
C7—H7	0.9500	C36—H36	0.9500
C8—C9	1.388 (8)	C37—H37	0.9500
C8—H8	0.9500	C38—C39	1.379 (7)
C9—C10	1.383 (9)	C38—C43	1.392 (7)
C9—C12	1.493 (8)	C38—P3	1.819 (5)
C10—C11	1.375 (8)	C39—C40	1.389 (8)
C10—H10	0.9500	C39—H39	0.9500
C11—H11	0.9500	C40—C41	1.381 (9)
C12—H12A	0.9800	C40—H40	0.9500
C12—H12B	0.9800	C41—C42	1.362 (9)
C12—H12C	0.9800	C41—H41	0.9500
C13—C14	1.377 (7)	C42—C43	1.394 (8)
C13—C18	1.378 (7)	C42—H42	0.9500
C13—P1	1.840 (5)	C43—H43	0.9500
C14—C15	1.390 (7)	C44—C49	1.381 (8)
C14—H14	0.9500	C44—C45	1.391 (7)
C15—C16	1.380 (8)	C44—P3	1.828 (5)
C15—H15	0.9500	C45—C46	1.366 (8)
C16—C17	1.371 (8)	C45—H45	0.9500
C16—H16	0.9500	C46—C47	1.372 (9)
C17—C18	1.385 (7)	C46—H46	0.9500
C17—H17	0.9500	C47—C48	1.367 (9)
C18—H18	0.9500	C47—H47	0.9500
C19—C20	1.370 (8)	C48—C49	1.360 (7)
C19—C24	1.387 (7)	C48—H48	0.9500
C19—P1	1.822 (5)	C49—H49	0.9500
C20—C21	1.385 (8)	C50—C55	1.384 (7)
C20—H20	0.9500	C50—C51	1.385 (7)
C21—C22	1.366 (10)	C50—P3	1.834 (5)
C21—H21	0.9500	C51—C52	1.357 (7)
C22—C23	1.349 (11)	C51—H51	0.9500
C22—H22	0.9500	C52—C53	1.377 (9)
C23—C24	1.375 (8)	C52—H52	0.9500
C23—H23	0.9500	C53—C54	1.356 (9)
C24—H24	0.9500	C53—H53	0.9500
C25—P1	1.837 (5)	C54—C55	1.382 (8)
C25—P2	1.839 (5)	C54—H54	0.9500
C25—H25A	0.9900	C55—H55	0.9500
C25—H25B	0.9900	Fe—P1	2.2389 (14)
C26—C31	1.379 (7)	Fe—Pt	2.5555 (9)
C26—C27	1.392 (7)	O4—S	1.432 (4)
C26—P2	1.827 (5)	O5—S	1.424 (4)
C27—C28	1.382 (8)	P2—Pt	2.3278 (13)
C27—H27	0.9500	P3—Pt	2.2680 (13)
			
O1—C1—Fe	176.6 (5)	C34—C35—H35	119.9
O2—C2—Fe	178.7 (5)	C37—C36—C35	119.6 (5)
O3—C3—Fe	176.2 (5)	C37—C36—H36	120.2
N1—C4—Pt	139.4 (4)	C35—C36—H36	120.2
N1—C4—Fe	140.7 (4)	C36—C37—C32	120.5 (5)
Pt—C4—Fe	79.6 (2)	C36—C37—H37	119.7
N1—C5—S	112.0 (3)	C32—C37—H37	119.7
N1—C5—H5A	109.2	C39—C38—C43	118.8 (5)
S—C5—H5A	109.2	C39—C38—P3	118.5 (4)
N1—C5—H5B	109.2	C43—C38—P3	122.4 (4)
S—C5—H5B	109.2	C38—C39—C40	120.4 (5)
H5A—C5—H5B	107.9	C38—C39—H39	119.8
C11—C6—C7	120.7 (5)	C40—C39—H39	119.8
C11—C6—S	119.3 (4)	C41—C40—C39	120.4 (6)
C7—C6—S	119.8 (4)	C41—C40—H40	119.8
C8—C7—C6	119.3 (5)	C39—C40—H40	119.8
C8—C7—H7	120.3	C42—C41—C40	119.5 (6)
C6—C7—H7	120.3	C42—C41—H41	120.2
C7—C8—C9	120.7 (6)	C40—C41—H41	120.2
C7—C8—H8	119.6	C41—C42—C43	120.7 (5)
C9—C8—H8	119.6	C41—C42—H42	119.7
C10—C9—C8	119.0 (5)	C43—C42—H42	119.7
C10—C9—C12	121.1 (6)	C38—C43—C42	120.1 (5)
C8—C9—C12	119.9 (6)	C38—C43—H43	120.0
C11—C10—C9	121.1 (6)	C42—C43—H43	120.0
C11—C10—H10	119.5	C49—C44—C45	117.9 (5)
C9—C10—H10	119.5	C49—C44—P3	119.9 (4)
C10—C11—C6	119.1 (5)	C45—C44—P3	122.1 (4)
C10—C11—H11	120.4	C46—C45—C44	119.8 (6)
C6—C11—H11	120.4	C46—C45—H45	120.1
C9—C12—H12A	109.5	C44—C45—H45	120.1
C9—C12—H12B	109.5	C45—C46—C47	120.8 (6)
H12A—C12—H12B	109.5	C45—C46—H46	119.6
C9—C12—H12C	109.5	C47—C46—H46	119.6
H12A—C12—H12C	109.5	C48—C47—C46	120.2 (5)
H12B—C12—H12C	109.5	C48—C47—H47	119.9
C14—C13—C18	119.8 (5)	C46—C47—H47	119.9
C14—C13—P1	123.4 (4)	C49—C48—C47	119.1 (6)
C18—C13—P1	116.8 (4)	C49—C48—H48	120.4
C13—C14—C15	119.7 (5)	C47—C48—H48	120.4
C13—C14—H14	120.2	C48—C49—C44	122.2 (5)
C15—C14—H14	120.2	C48—C49—H49	118.9
C16—C15—C14	120.0 (5)	C44—C49—H49	118.9
C16—C15—H15	120.0	C55—C50—C51	118.5 (5)
C14—C15—H15	120.0	C55—C50—P3	121.9 (4)
C17—C16—C15	120.3 (5)	C51—C50—P3	119.6 (4)
C17—C16—H16	119.8	C52—C51—C50	120.8 (5)
C15—C16—H16	119.8	C52—C51—H51	119.6
C16—C17—C18	119.5 (5)	C50—C51—H51	119.6
C16—C17—H17	120.2	C51—C52—C53	120.7 (5)
C18—C17—H17	120.2	C51—C52—H52	119.7
C13—C18—C17	120.6 (5)	C53—C52—H52	119.7
C13—C18—H18	119.7	C54—C53—C52	119.3 (5)
C17—C18—H18	119.7	C54—C53—H53	120.3
C20—C19—C24	118.8 (5)	C52—C53—H53	120.3
C20—C19—P1	121.4 (4)	C53—C54—C55	120.9 (5)
C24—C19—P1	119.7 (4)	C53—C54—H54	119.6
C19—C20—C21	120.4 (6)	C55—C54—H54	119.6
C19—C20—H20	119.8	C54—C55—C50	119.9 (5)
C21—C20—H20	119.8	C54—C55—H55	120.1
C22—C21—C20	119.5 (6)	C50—C55—H55	120.1
C22—C21—H21	120.2	C2—Fe—C1	94.8 (3)
C20—C21—H21	120.2	C2—Fe—C3	98.2 (2)
C23—C22—C21	121.0 (6)	C1—Fe—C3	165.4 (2)
C23—C22—H22	119.5	C2—Fe—C4	106.5 (2)
C21—C22—H22	119.5	C1—Fe—C4	82.6 (2)
C22—C23—C24	120.0 (6)	C3—Fe—C4	87.3 (2)
C22—C23—H23	120.0	C2—Fe—P1	109.51 (16)
C24—C23—H23	120.0	C1—Fe—P1	92.75 (17)
C23—C24—C19	120.4 (6)	C3—Fe—P1	89.22 (16)
C23—C24—H24	119.8	C4—Fe—P1	143.95 (17)
C19—C24—H24	119.8	C2—Fe—Pt	154.45 (16)
P1—C25—P2	115.4 (3)	C1—Fe—Pt	91.70 (18)
P1—C25—H25A	108.4	C3—Fe—Pt	73.70 (16)
P2—C25—H25A	108.4	C4—Fe—Pt	49.91 (16)
P1—C25—H25B	108.4	P1—Fe—Pt	94.81 (4)
P2—C25—H25B	108.4	C4—N1—C5	121.1 (4)
H25A—C25—H25B	107.5	C19—P1—C25	99.2 (2)
C31—C26—C27	118.6 (5)	C19—P1—C13	101.1 (2)
C31—C26—P2	117.9 (4)	C25—P1—C13	105.7 (2)
C27—C26—P2	123.5 (4)	C19—P1—Fe	118.40 (18)
C28—C27—C26	120.2 (5)	C25—P1—Fe	111.74 (15)
C28—C27—H27	119.9	C13—P1—Fe	118.27 (16)
C26—C27—H27	119.9	C26—P2—C32	103.6 (2)
C29—C28—C27	120.5 (6)	C26—P2—C25	100.9 (2)
C29—C28—H28	119.8	C32—P2—C25	103.8 (2)
C27—C28—H28	119.8	C26—P2—Pt	122.38 (16)
C28—C29—C30	120.0 (5)	C32—P2—Pt	115.77 (16)
C28—C29—H29	120.0	C25—P2—Pt	107.91 (16)
C30—C29—H29	120.0	C38—P3—C44	101.9 (2)
C29—C30—C31	120.4 (5)	C38—P3—C50	102.1 (2)
C29—C30—H30	119.8	C44—P3—C50	105.2 (2)
C31—C30—H30	119.8	C38—P3—Pt	120.51 (16)
C26—C31—C30	120.3 (5)	C44—P3—Pt	111.37 (18)
C26—C31—H31	119.8	C50—P3—Pt	113.99 (16)
C30—C31—H31	119.8	C4—Pt—P3	97.70 (14)
C37—C32—C33	119.2 (5)	C4—Pt—P2	148.96 (14)
C37—C32—P2	119.2 (4)	P3—Pt—P2	112.97 (5)
C33—C32—P2	121.5 (4)	C4—Pt—Fe	50.45 (14)
C34—C33—C32	120.7 (5)	P3—Pt—Fe	146.99 (4)
C34—C33—H33	119.7	P2—Pt—Fe	98.51 (4)
C32—C33—H33	119.7	O5—S—O4	119.0 (2)
C33—C34—C35	119.9 (5)	O5—S—C6	108.1 (2)
C33—C34—H34	120.1	O4—S—C6	108.8 (2)
C35—C34—H34	120.1	O5—S—C5	108.6 (2)
C36—C35—C34	120.2 (5)	O4—S—C5	105.6 (2)
C36—C35—H35	119.9	C6—S—C5	106.0 (2)

## References

[bb1] Basset, J. M., Barker, G. K., Green, M., Howard, J. A. K., Stone, F. G. A. & Wolsey, W. C. (1981). *J. Chem. Soc. Dalton Trans.* pp. 219–227.

[bb2] Chen, P., Peng, Y., Jia, C. & Qu, J. (2010). *Eur. J. Inorg. Chem.* pp. 5239–5246.

[bb23] Farrugia, L. J. (1997). *J. Appl. Cryst.* **30**, 565.

[bb3] Farrugia, L. J. (1999). *J. Appl. Cryst.* **32**, 837–838.

[bb4] Fontaine, X. L. R., Jacobsen, G. B., Shaw, B. L. & Thornton-Pett, M. (1988). *J. Chem. Soc. Dalton Trans.* pp. 741–750.

[bb5] Knorr, M., Faure, T. & Braunstein, P. (1993). *J. Organomet. Chem.* **447**, C4–C6.

[bb6] Knorr, M., Jourdain, I., Crini, G., Frank, K., Sachdev, H. & Strohmann, C. (2002). *Eur. J. Inorg. Chem.* pp. 2419–2426.

[bb7] Knorr, M., Jourdain, I., Lentz, D., Willemsen, S. & Strohmann, C. (2003). *J. Organomet. Chem.* **684**, 216–229.

[bb8] Knorr, M. & Strohmann, C. (2000). *Eur. J. Inorg. Chem.* pp. 241–252.

[bb9] Ratliff, K. S., Fanwick, P. E. & Kubiak, C. P. (1990). *Polyhedron*, **9**, 2651–2653.

[bb10] Sheldrick, G. M. (2008). *Acta Cryst.* A**64**, 112–122. 10.1107/S0108767307043930 18156677

[bb11] Stoe & Cie (1999). *IPDS* Stoe & Cie, Darmstadt, Germany.

